# A local sequence alignment approach to recognizing fixed poetic forms across languages

**DOI:** 10.1371/journal.pone.0340514

**Published:** 2026-07-14

**Authors:** Petr Plecháč, Artjoms Šeļa

**Affiliations:** 1 Institute of Czech Literature, Czech Academy of Sciences, Prague, Czechia; 2 University of Tartu, Tartu, Estonia; Indiana University Bloomington, UNITED STATES OF AMERICA

## Abstract

Fixed poetic forms such as the sonnet, ottava rima, or terza rima are an important feature of European literary traditions, yet large-scale empirical research on their cross-lingual distribution and evolution has been limited so far. This paper introduces a fully language-independent, unsupervised method for identifying recurrent rhyme-based forms using local sequence alignment. Drawing on 187,719 poems from six European traditions (Czech, English, French, German, Italian, Russian) in the PoeTree collection, we encode rhyme schemes in a compact eight-symbol alphabet and apply the Smith–Waterman algorithm via the Metronome package to compute pairwise distances. Dimensionality reduction (UMAP) and density-based clustering (HDBSCAN) yield 61 clusters, many of which align with known fixed forms. Evaluation against existing Czech and Russian annotations shows strong recall, while supervised classification experiments—both within and across languages—demonstrate that form categories are robustly learnable in the induced vector space. We illustrate the potential of such data for literary research in three showcases: cross-tradition influence in 19th-century Czech poetry, topical affinities of selected forms using multilingual topic modeling, and geographic associations revealed through geonym analysis.

## Introduction

Fixed forms—such as the sonnet, terza rima, or the Sicilian octave—are among the most distinctive features of European poetic traditions. Defined primarily by finite or recurrent rhyme schemes, which are largely independent of language, these forms readily crossed the boundaries between traditions, while often preserving associations with their earlier uses. Once thus an experienced reader encounters fourteen lines of sonnet regardless of the language, they anticipate the articulation of love, stanzas in the *abababcc* scheme of the ottava rima are likely to suggest heroic narrative (or its parody), and terza rima will almost inevitably recall Dante’s Divine Comedy and, by extension, signal the grand tradition of Italian Renaissance.

Systematic cross-language empirical studies of fixed forms use and evolution, however, remain scarce. Existing research have often been limited to single traditions (e.g., [1,2]) or a synthesis of their findings [3]. Poetry datasets that could support large-scale research—when they include information on fixed forms at all—tend to rely either on manual annotation [4,5] or on relatively simple rule-based algorithms [6]. While the former is prone to inconsistencies—especially when multiple annotators are involved—the latter risks overlooking relevant instances whenever a form appears with even slight variation. A recent study [7] reports reasonably good accuracy in fixed-form recognition by several large language models, but even this approach risks imposing prescriptive rules—that the model may have memorized from poetic manuals—and overlooking important variation.

In this paper, we propose a language-independent unsupervised method for the recognition of fixed forms—or more broadly rhyme patterns that have been reused over time and traditions. Instead of relying on expert assumption or their embedding into a set of rules, we focus on regularities emerging from the data itself using local sequence alignment, an approach previously shown to be effective in the analysis of poetic meter [8]. Our goal is to identify groups of poems that share similar rhyme schemes across six European poetic traditions, namely Czech, English, French, German, Italian, and Russian. These not only cover each of the three major typological branches of European languages—Germanic (English, German), Romance (French, Italian), and Slavic (Czech, Russian)—but also both major versification systems found in modern European poetry, i.e., syllabic (French, Italian) and accentual-syllabic (Czech, English, German, Russian).

To demonstrate the potential application of the method to questions of comparative literary history, we present three showcases, focusing on: (1) the diffusion and adoption of international forms; (2) associations between forms and themes across traditions; and (3) the use of forms and their imaginary geography.

The first showcase focuses on transnational literary dynamics. By comparing traditions as frequency distributions of poetic forms over time, we show how a minor national literature (Czech), over the course of the nineteenth century, becomes increasingly similar to the European literary field, which itself grows more homogeneous.

The second showcase addresses one of the fundamental questions in verse studies: the relationship between form and meaning. Traditionally, this problem has been discussed primarily in relation to meter [9,10]. However, it is increasingly clear that poetic form—particularly the rhyme organization of the stanza—maintains strong semantic affinities and is especially important in cases of translation across different versification systems [11] (e.g., from syllabic Spanish to accentual-syllabic Russian). Building on recent large-scale confirmation of persistent associations between form and meter in European verse [12], we use cross-lingual topic modeling to show that certain stanzaic and fixed forms, in turn, share thematic affinities that persist across languages. This provides clear evidence of a shared organizing tradition underlying their use, one that is not confined to individual literatures.

The third showcase extends the question of form and meaning to the geographic locations referenced within distinct forms. By examining the distribution of geonyms across different stanza types, we show how certain common forms, such as the ABAB quatrain, become naturalized within a national tradition, while others, such as terza rima, retain a connection to their origin in Italy and a strong association with Dante, thus serving as vehicles for literary-historical imagination.

## Data & methods

All poems analyzed in this study are drawn from the PoeTree collection [13]. Although the corpus contains over 270,000 texts in the six languages under consideration, we restrict our analysis to poems in which no stanza exceeds ten lines, as longer stanzas make the task computationally prohibitive. Even with this constraint, the dataset remains large enough, comprising 187,719 texts: approximately 56k in Czech, 35k in German, 25k in English, 13k in French, 28k in Italian, and 31k in Russian.

Our method builds on the availability of pre-annotated rhymes. Where possible, we draw on existing resources, namely the Corpus of Czech Verse [6] for Czech, Metricalizer [14] for German, and Malherbe [15] for French. For English, Italian, and Russian, rhyme annotation was performed with the rhymeTagger package [16], whose accuracy in these languages has been shown to be satisfactory [17]. To reduce the risk of spurious matches, we impose a limit on rhyme span: any pair of rhymes separated by more than six intervening lines is disregarded.

### Rhyme encoding

In the conventional rhyme notation, lines that rhyme share the same letter of the alphabet, while *x* is reserved for unrhymed lines. Thus the opening two quatrains of *The Rime of the Ancient Mariner*:

It is an ancient Mariner,

And he stoppeth one of three.

’By thy long grey beard and glittering eye,

Now wherefore stopp’st thou me?

The Bridegroom’s doors are opened wide,

And I am next of kin;

The guests are met, the feast is set:

May’st hear the merry din.’

would be encoded as *xaxa xbxb*. For our purposes, however, this encoding is brittle. Not only can one easily run out of letters with a poem of only few dozen lines, but, more importantly, a single error in rhyme recognition can cascade and misalign the entire scheme. Consider two Shakespearean sonnets with the same scheme (*abab cdcd efef gg*). If the rhyme between the first and third lines is missed in the second sonnet, the derived scheme becomes *xaxa bcbc dede ff*, leaving no symbol-level matches with the correct representation. Instead we employ an ad hoc encoding which overcomes these issues at the cost of lowered readability. Each line is still represented by a single letter, yet they encode how many lines further in the text their rhyming counterpart is found: *b* stands for immediately following line, *c* stands for line that is one step apart, *d* for two steps apart..., *x* is reserved for those having no rhyming counterpart that *follows* them in the text, and full stop marks the stanza boundaries. Correct encoding of Shakespearean sonnet in our notation thus becomes *ccxx.ccxx.ccxx.bx.*:

**Table:** 

	conv.	ad hoc
Shall I compare thee to a summer’s day?	*a*	*c*
Thou art more lovely and more temperate:	*b*	*c*
Rough winds do shake the darling buds of May,	*a*	*x*
And summer’s lease hath all too short a date:	*b*	*x*
		.
Sometime too hot the eye of heaven shines,	*c*	*c*
And often is his gold complexion dimm’d;	*d*	*c*
And every fair from fair sometime declines,	*c*	*x*
By chance or nature’s changing course untrimm’d;	*d*	*x*
		.
But thy eternal summer shall not fade,	*e*	*c*
Nor lose possession of that fair thou ow’st;	*f*	*c*
Nor shall death brag thou wander’st in his shade,	*e*	*x*
When in eternal lines to time thou grow’st:	*f*	*x*
		.
So long as men can breathe or eyes can see,	*g*	*b*
So long lives this, and this gives life to thee.	*g*	*x*

When the first rhyme is misrecognized it becomes *xcxx.ccxx.ccxx.bx.*, which leaves only one symbol-level mismatch. Given the limit on maximum rhyme span, our alphabet for rhyme encoding consists of 8 symbols only (*b, c, d, e, f, g, h* for rhymes being 0–6 steps apart, *x* for nonrhyming lines and*.* for stanza boundaries). For the sake of readability, however, we will use the conventional rhyme notation in examples and illustrations further in this paper.

### Forms detection

All poems encoded in the 8-symbol alphabet are subject to local sequence alignment. We use the Metronome package [18], which applies the Smith–Waterman algorithm as implemented in Biopython [19] to text processing. Local sequence alignment is preferred here over alternatives such as edit distance or bag-of-symbols representations: edit distance treats every substitution, insertion, or deletion equally across the full string, penalizing length variation between poems rather than formal difference, whereas a bag-of-symbols approach discards positional information entirely, collapsing structurally distinct schemes that share identical symbol frequencies (e.g., abba vs. abab). Local alignment, by contrast, rewards shared subsequences wherever they occur within the string, making it robust to length variation and partial formal similarity. This procedure yields a 187,719×187,719 distance matrix.

**Fig 1:**
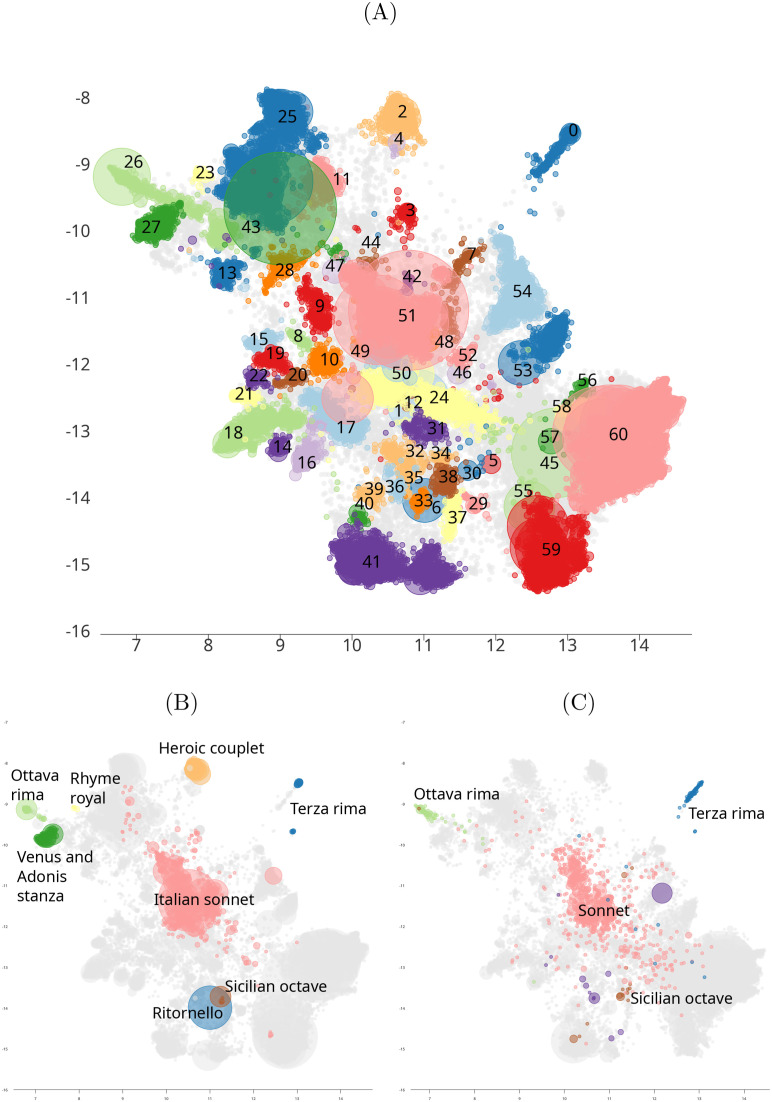
UMAP 2-dimensional projections of rhyme schemes. (A) HDBSCAN clustering of poems from six corpora (PoeTree.[cs, de, en, fr, it, ru]). (b) PoeTree.cs: prelabeled forms. (C) PoeTree.ru: prelabeled forms.

We then reduce the dimensionality of the resulting vector space to 50 dimensions using the UMAP algorithm (n_neighbors = 20, min_dist = 0). UMAP is preferred over alternatives such as PCA or t-SNE because it preserves both local and global structure simultaneously, making the reduced space suitable both for subsequent clustering and for visualization. We subsequently perform clustering with HDBSCAN (min_cluster_size = 100, min_samples = None]. HDBSCAN is chosen because it requires no prior specification of the number of clusters and because it explicitly models noise, assigning poems that match no recurring pattern to an outlier class rather than forcing them into the nearest cluster. This procedure groups the poems across corpora into 61 clusters, with additional outliers. The results, after a further UMAP reduction to two dimensions (n_neighbors = 20, min_dist = 0), are visualized in Fig 1A.

As an initial means of orientation within this plot, we compare these clusters with the same space visualization of fixed forms labels available for two of the corpora, namely Czech where it has been produced by a rule-based algorithm [6] in Fig 1B, and Russian where the annotation was done manually [4] in Fig 1C; we disregard any forms having less than 50 instances in a given corpus as well as the annotation available for Italian [5] as it largely comprise theme-defined forms, such as the eclogue or lauda.

At first glance, the clusters produced by HDBSCAN far outnumber the fixed forms annotated in either corpus. This is hardly surprising, since the algorithm groups together any recurring pattern, including relatively trivial ones such as alternating-rhyme quatrains (abab cdcd…; cluster 60), paired-rhyme quatrains (aabb ccdd…; cluster 25), or other recurrent configurations that happened not to be coined in traditional poetics. All prelabeled forms, however, generally align with specific clusters. In both corpora, sonnet largely corresponds to cluster 51, terza rima to cluster 0, ottava rima to cluster 26, and Sicilian octave to cluster 38. In the Czech corpus, heroic couplet maps onto cluster 2, rhyme royal onto cluster 23, Venus&Adonis onto cluster 27, and ritornello onto cluster 6. On the other hand, there seem to be a general trend (most apparent in Czech data): with the exception of sonnet, clusters tend to extend well beyond the regions occupied by their prelabeled counterparts. It is difficult to tell to what extent this represents genuine variation overlooked by the labels, and to what extent it reflects unwanted noise. This is something to keep in mind during evaluation.

## Evaluation

### Prelabeled forms

To begin, we evaluate all Czech and Russian data, treating the prelabeled forms as the gold standard. For each form, we identify the cluster with the largest overlap and compute its precision and recall. The results (Table 1) are somewhat inconsistent.

**Table 1:** Precision and recall of clustering-based recognition evaluated against prelabeled forms.

prelabeled form	cluster	CS			RU		
		# of poems	precision	recall	# of poems	precision	recall
(Italian) sonnet	51	5568	0.87	0.62	1321	0.55	0.30
Venus and Adonis stanza	27	741	0.79	0.82	–	–	–
Ritornello	6	655	0.997	1	–	–	–
Ottava rima	26	214	0.32	0.99	92	0.25	0.89
Heroic couplet	2	209	0.12	1	–	–	–
Sicilian octave	38	135	0.53	1	–	–	–
Terza rima	0	54	0.32	0.85	147	0.9	0.93

*Sonnet*. The most frequent form in both corpora is also the only one for which precision exceeds recall. This likely reflects the breadth of the sonnet category: virtually any 14-line poem tends to be annotated as a sonnet, regardless of rhyme scheme. As a result, sonnet-labeled poems are spread across several clusters—most notably cluster 51 (abba abba octave), cluster 50 (abab abab octave), and even cluster 54, which contains unrhymed poems.

*Venus & Adonis, ritornello, terza rima*. These three forms achieve relatively high values on both metrics, with one exception: Czech terza rima, whose precision is only 0.32. As Fig 1 already suggests, this is likely due to overly restrictive annotation rules. Russian manual labels span roughly the same region as the corresponding cluster, whereas Czech rule-based labels fail to capture even the canonical variant [20, 1423] in which the poem ends with a couplet rather than a single-line stanza. This is futher supported by self-references: 4 of the 16 poems whose title contains “terc[ií]n.*” (the Czech term for terza rima) are not labeled as such in the Czech data.

*Ottava rima, heroic couplet, Sicilian octave*. The remaining forms exhibit a similar pattern to Czech terza rima: solid precision but poor recall.

At first glance, our precision values fall well below those reported for LLM-driven recognition. This gap, however, is largely an artifact of how the two approaches are evaluated. The dataset of [7] contains only poems with an assigned form label, while in our case 86% of Czech poems and 95% of Russian poems are unlabeled in the gold standard. When we restrict the evaluation to poems the gold standard does recognize—the only fair basis for comparison—precision increases dramatically. The only value below 0.99 is Czech ottava rima (precision 0.62), where the corresponding cluster is contaminated by 126 poems the gold standard labels as Venus&Adonis. The reason is straightforward: all of these misclassified poems consist of a single stanza, and in such case Venus&Adonis scheme (ababcc) is a substring of that of ottava rima (abababcc), resulting in effectively zero distance between the two under local sequence alignment.

On this restricted evaluation, our results are thus broadly comparable to those of LLM-based recognition, with the caveat that full comparability is limited by differences in tagsets. The picture changes when evaluation is extended to all poems: precision drops considerably, suggesting a high false-positive rate. Whether these apparent errors reflect genuine misclassifications by our method or systematic gaps in the gold standard cannot be determined from the evidence available. The relatively high silhouette coefficient (*SC* = 0.49), however, indicates that the clustering itself is meaningful.

### Supervised learning

To further test our approach in a gold-positive scenario we train a set of classifiers to predict form labels. For this task, we include all the forms represented by at least 15 instance in Czech or Russian corpus, which broadens the tagset to 9 forms in Czech (Italian sonnet, Venus&Adonis, ritornello, ottava rima, heroic couplet, Sicilian octave, terza rima, English sonnet, Limerick) and 5 forms in Russian (sonnet, ottava rima, terza rima, Sicilian octave, triolet). In addition, drawing on several prosody manuals, we manually curated a set of 231 instances belonging to one of the following forms in English corpus: sonnet, terza rima, triolet, ottava rima, and rhyme royal.

Each poem is represented as a vector in the 50-dimensional space obtained from the first UMAP reduction (Introduction). We then downsample to 15 instances per class and perform leave-one-out validation within the corpus using three classifiers: Random Forest, Support Vector Machine, and k-nearest neighbors (*k* = 5). These three classifiers were selected because they represent qualitatively distinct inductive biases—ensemble tree-based, margin-based, and instance-based learning respectively—such that consistent performance across all three would indicate that the signal in the vector space is robust and not an artifact of any particular classifier’s assumptions. The downsampling and validation procedure is repeated 100 times, resulting in 13,500 classifications in Czech, and 7,500 classifications in Russian and English.

Across all three languages, the random forest classifier consistently achieves the best performance, although the other two classifiers are not far behind (Table 2). For Czech, it reaches an accuracy of 0.97, with most errors occurring between the English and Italian sonnet types. In English and Russian, accuracy decreases despite the smaller number of classes, yet remains at a solid level; in both cases, most misclassifications involve confusion between the sonnet and the triolet.

**Table 2:** Leave-one-out cross-validation results for supervised classification of pre-labeled forms.

	SVM	RF	KNN
Czech	0.95	0.97	0.93
English	0.91	0.93	0.92
Russian	0.90	0.93	0.88

Support Vector Machine (SVM), Random Forest (RF), *K*-nearest neighbors (KNN).

We also evaluate classification in a cross-language scenario. For each pair of the three languages, we select only the verse forms shared by both. If we somewhat incorrectly treat the “Italian sonnet” category in Czech as equivalent to the broader “sonnet” category in English and Russian (noting that in the former the English type is actually likely to dominate), this results in four shared forms for the (Czech, Russian) and (English, Russian) pairs, and three forms for the (Czech, English) pair. We then downsample each class to 15 instances and successively train the classifiers on one language, testing them on the remaining two. Once again, the entire procedure is repeated 100 times.

Even in the cross-language scenario, accuracy remains well above 0.9, with no clear winner among the classifiers (Table 3). The only exception is the English–Russian pair, in both directions, where confusion between the sonnet and the triolet becomes even more pronounced. We assume this is caused by likely prevalence of the Italian sonnet type in Russian and the English sonnet type in English.

**Table 3:** Leave-one-out cross-validation results for cross-language supervised classification of pre-labeled forms.

training	test	SVM	RF	KNN
Czech	English	0.95	0.95	0.95
	Russian	0.92	0.92	0.91
English	Czech	0.91	0.93	0.997
	Russian	0.87	0.84	0.92
Russian	Czech	0.97	0.98	0.96
	English	0.78	0.79	0.78

Support Vector Machine (SVM), Random Forest (RF), *K*-nearest neighbors (KNN).

We have shown that clustering in the vector space derived from local sequence alignment generally yields solid recall values in two of the corpora. Although precision remains rather low, this does not necessarily signal contamination of clusters with irrelevant material; rather, it may reflect overly narrow category boundaries in the default annotation itself. Moreover, our experiments with supervised learning indicate that these default definitions are, to a large extent, easy to replicate in our representations of poetic texts. In the following sections, we present three showcases that illustrate how form-annotated corpora can support literary research.

## Showcases

### Form distribution: Czech and the major traditions

At the beginning of the nineteenth century—a period from which a majority of our data is drawn—Czech poetry was in a markedly different situation from the major European literary traditions such as German, English, French, and Russian, as it was only beginning to re-emerge after more than a century of decline. In this stage, Czech literature tended to mimic and adopt from stronger traditions. Since fixed forms—as already mentioned—can traverse the language boundaries with ease, we ask whether this process can be traced in their domain as well.

We include all authors from the Czech, German, English, French, and Russian corpora born between 1775 and 1900 (excluding Italian, for which data from this period are sparse), and group them into 25-year birth cohorts. Since we assume that the transfer of forms from one tradition to another occurs with some delay, we focus primarily on situations when poetry of one generation is becoming more similar, in terms of forms, to that of the previous generation in different tradition than to the preceding generation of its own tradition. To model this, we represent each cohort within each corpus as a vector defined by the relative frequencies of 61 HDBSCAN-derived forms in the cohort’s works. For each non-initial cohort in each language, we measure the cosine similarity between its vector and that of the preceding cohort in the same language, as well as its similarities to preceding cohorts in other languages. Whenever a cross-corpus similarity exceeds the intra-corpus one, we consider this an influence effect and measure its size as the simple difference between the two values. At the same time, we also measure the cosine similarity between concurrent cohorts across languages to model how similar the traditions are to each other at each stage.

**Fig 2:**
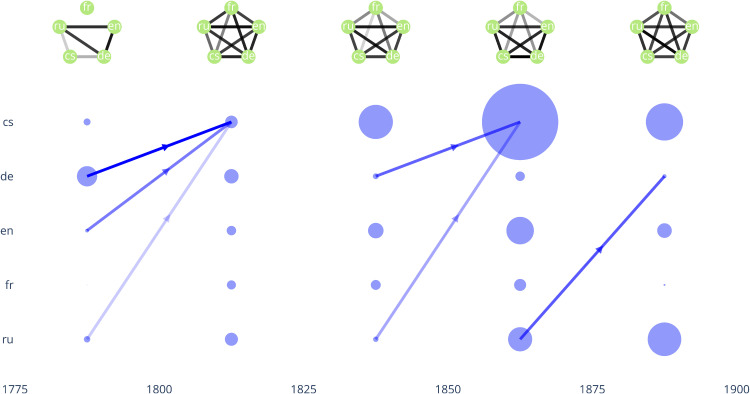
Form distribution similarity. Each bubble represents authors born within a 25-year time span; its size corresponds to the number of poems. Edges represent influence effect, with thickness proportional to its size. The pentagrams at the top of the chart show the degree of similarity between concurrent cohorts.

The results (Fig 2) corroborate our hypothesis. Among the earliest cohorts (1775–1800), there is already a high degree of similarity between the major traditions–German, English and Russian (no French data for this period)—while Czech remains only loosely connected. In the following stage (1800–1825), Czech poetry assimilates into the major traditions, showing greater similarity to the eariler stages of German, English and, to a lesser extent, Russian poetry—than to its preceding stage. Note that beside 1875–1900 German cohort, where it may be due to insufficient data, it is only in the Czech corpus, here and again two stages later, where such effect appears.

### Topic modeling: quatrains, sonnets, and quatrain-chains

The second showcase stems from the assumption outlined in Introduction, namely that fixed forms tend to preserve the thematic contexts of their earlier uses. To test this, we trained a multilingual topic model using Paraphrase sentence embeddings [21] and BERTopic [22], resulting in each poem being represented as a probability distribution over 207 topics.

We then selected three form-clusters that are both frequent across corpora and likely to exhibit thematic or genre associations:

- cluster 45 (single quatrain with *abab* scheme): primarily associated with epigrams [20, 449–450, 1138–1139];
- cluster 51 (Petrarchan sonnet): lyrical expression of emotions, though later broadening in scope [20, 1318–1321];
- cluster 60 (multiple quatrains with *abab* scheme): rather theme-agnostic, but often linked to folklore, ballads and songs, rural settings, and depictions of nature [20, 114–119, 1138–1139].

**Fig 3:**
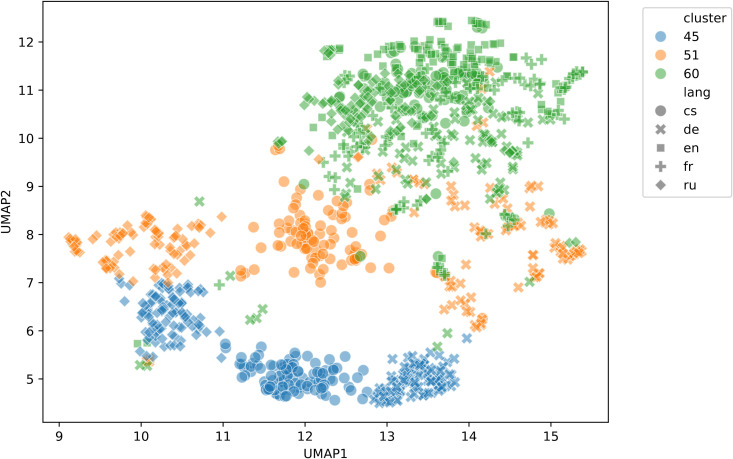
UMAP projection of single *abab* quatrain samples (45), sonnet samples (51), and *abab* quatrain-chains (60) into topic defined vector space.

To model the general semantic associations of these forms we draw a 100 random samples of 100 poems (with replacement) from each corpus, where they have at least 500 instances and project each sample into the 207-dimensional vector space defined by its average topic probabilities, transformed to *z*-scores. Fig 3 shows that the relations of three forms exhibit a strong topical similarity across languages. In the upper region, we observe a cluster of quatrain-chain samples from Czech, German, English, French, and Russian, with only a few outliers. Czech, German, and Russian sonnet samples are located in the central area, while single-quatrain samples form a distinct cluster in the lower region.

To peek into which topics are most decisive for each form, we trained a random forest classifier (achieving 91% accuracy in leave-one-out cross validation) and extracted Shapley values (see S1 Fig, S2 Fig, and S3 Fig). For single quatrains, two topics clearly stand out—humor (100) and theft (184)—both supporting the epigram hypothesis raised above. Sonnets, by contrast, draw on a much broader thematic field, but generally lean toward highbrow motifs such as stars (84), death (123), high art (59), or urban life (85). Quatrain-chains lean more toward rural themes, including the seasons (46), forests (168), and dancing (108). Somewhat surprisingly, it is here—and not in the sonnet—that the love topic (179) comes to the fore. Our provisional explanation is that in quatrains it tends to be expressed more directly and is thus easier for the model to capture, whereas in sonnets it is often framed in more periphrastic or metaphorical ways. As one of the central lyrical forms in European poetry, sonnet also extended its thematic range with the transformation of the lyrical voice; as the topic model suggests, the form is defined through existential and contemplative themes linked to urban modernity.

### Geographic references

In the last showcase, we focused briefly on the use of geonyms, basing on the assumption that fixed forms may vary in their degree of naturalization. We expect that some of them may retain associations with the traditions from which they were adopted, which in turn could manifest in higher frequencies of references to locations in the corresponding regions.

We employ the PoeTree geonyms dataset [23], which contains over 200,000 references to real-world locations identified in the respective corpora, along with their geographic coordinates. Based on these coordinates, we assign each location to its present-day country using Reverse Geocode [24]—remaining aware of limitations of such ahistorical approach—and measure the relative importance of individual countries for each poetic form within each corpus using TF-IDF.

**Fig 4:**
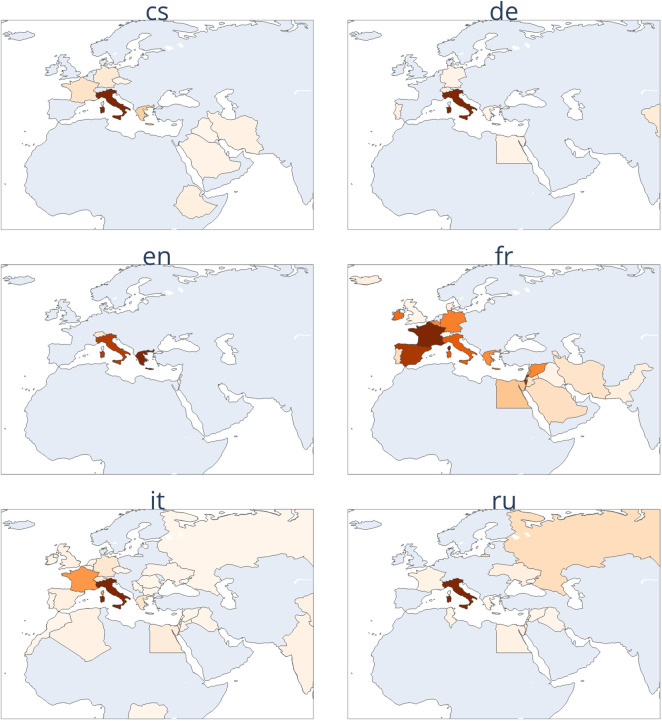
TF-IDF scores of individual country-references in cluster 0 (terza rima).

**Fig 5:**
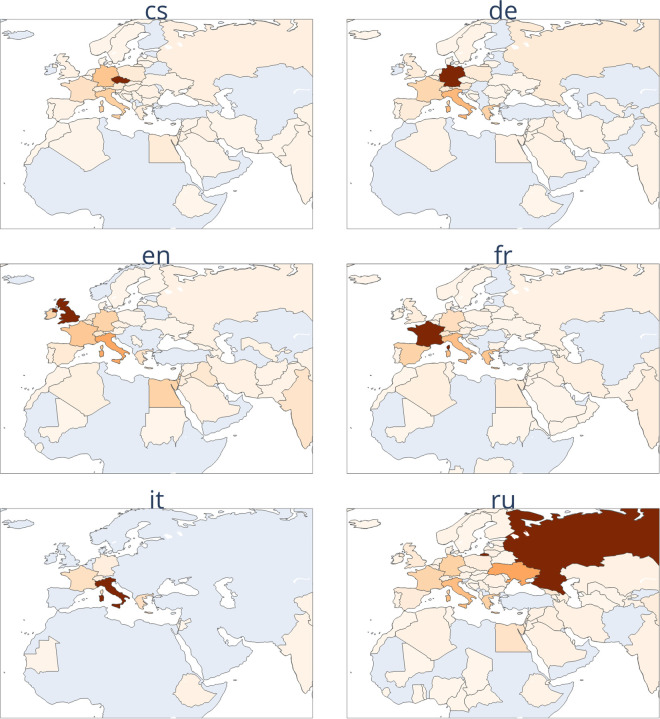
TF-IDF scores of individual country-references in cluster 60 (*abab* quatrain-chains).

While in most forms domestic references dominate, one country clearly stands out: Italy. Unsurprisingly, Italian references score highest across corpora in forms such as terza rima (0), ritornello (6), ottava rima (26), and in the various types of sonnet. Fig 4 and Fig 5 illustrate this by contrasting terza rima with the traditional *abab* quatrain chains. In terza rima, Italian references consistently reach the highest scores across corpora—with the exception of French, where domestic and Spanish references take precedence—whereas in quatrains domestic traditions dominate by far. Although this evidence is necessarily selective and somewhat anecdotal, it suggests this area is worth exploring further, ideally with a more historically sensitive toolkit and a diachronic perspective.

## Discussion

This study extends the use of local sequence alignment as applied to poetic structure by using it to identify recurrent rhyme-based forms across large multilingual corpora. By representing rhyme schemes in a compact symbolic alphabet and comparing them with the Smith–Waterman algorithm, our method captures similarities that conventional rule-based models or prescriptive labeling often miss. The resulting clusters align well with established fixed forms, but they also reveal a much larger variation of rhyme and stanza organizations than is normally considered in computational approach to poetic form.

We are able to show that: (1) Unsupervised clustering generally recovers groups corresponding to known forms. Low precision appear to reflect, at least in part, limitations of existing labels and the significant amount of formal variation present in historical data, rather than shortcomings in our approach. (2) Supervised classification—performed both within and across languages—demonstrates that the “stanzaic vector space” we construct is robust in identifying known classes.

The three showcases demonstrate how such representations open new possibilities for literary-historical inquiry. By aligning multiple languages within a shared formal space, we are able to trace the homogenization of poetic form in European poetry and the gradual incorporation of Czech poetry into a broader European network. Topic modeling confirms that fixed forms retain distinct thematic associations across languages, forming separate traditions. The geonym study similarly suggests that some forms preserve cultural and thematic localization: Italian-origin forms continue to exhibit disproportionately frequent references to Italy in non-Italian traditions, reflecting a long-term continuity in the modern perception and symbolic placement of the Italian Renaissance within European literatures.

Several limitations and directions for future research emerge from this work. First, our approach to sequence alignment can be further refined. Because we compare strings locally, variation in poem length becomes a significant factor. In some cases, long poems written in regular rhyme schemes become closely aligned with two- or four-line epigrams because an incorrectly detected rhyme in one stanza produces a fragment that shares more similarity with a short poem than with other long poems of similar structure. The design of the scoring matrix also warrants further evaluation, but this requires examining theoretical assumptions about stanza and form rather than simple optimization. For example, should visual stanza boundaries always be treated as structurally meaningful? If so, is a sonnet divided into four stanzas different from one written as fourteen consecutive lines? And can we trust stanza boundaries in large collections of digitized poetry spanning different periods and editions?

Second, stanzaic structure is not determined by rhyme scheme alone; it also results from the interaction between meter and rhyme [25]. Computational approaches to the history of poetic forms should therefore integrate both dimensions, as was routinely done in the Russian versification tradition, which encoded meter–rhyme units using specifically designed “metrical formulas” [4]—not unlike how a “floral formula” is used in botany to summarize and describe the structure of flowers.

Despite these limitations, our findings invite to adjust the concept of poetic “form.” The clusters suggest that many recurrent rhyme configurations fall outside canonical categories, yet still share structural affinities with them. This raises questions about continuity, innovation, and experimentation in poetic form. Does the fourteen-line Onegin stanza adapt the sonnet form in order to write a “novel in verse” to clash lyric and narrative traditions? Or is it instead a further development of Byron’s narrative octaves? Or perhaps an iteration of the ten-line ode stanza?

More generally, we lack good theories of the underlying historical processes at work. To what extent structural similarities in poetic form are driven by independent experimentation that converges on similar forms for different purposes, and to what extent do they emerge as distinct historical lineages that carry recognizable syntactic and cultural imprints through time? A sonnet may be a case of the latter, while some “odd stanza” [26]—a constellation of individual sense of form and phrase, never used frequently—a case of the former. By approaching form inductively rather than prescriptively, our method shifts attention from what poetic forms ought to be to what poets actually produced—and, in doing so, suggests a way of treating form less as a fixed category and more as a historically bounded continuity that transforms, restructures, and resurfaces as it passes between hands and generations.

## Supporting information

S1 Fig Cluster 45: single *abab*-quatrain. Shapley values in topic-defined vector space.
(TIFF)

S2 Fig Cluster 51: sonnet. Shapley values in topic-defined vector space.
(TIFF)

S3 Fig Cluster 60: *abab*-quatrain chains. Shapley values in topic-defined vector space.
(TIFF)
