# Supplementary figures and images for: A local sequence alignment approach to recognizing fixed poetic forms across languages
Source: PLoS One. 2026 Jul 14;21(7):e0340514. doi: 10.1371/journal.pone.0340514 (PMC13367689; doi:10.1371/journal.pone.0340514)

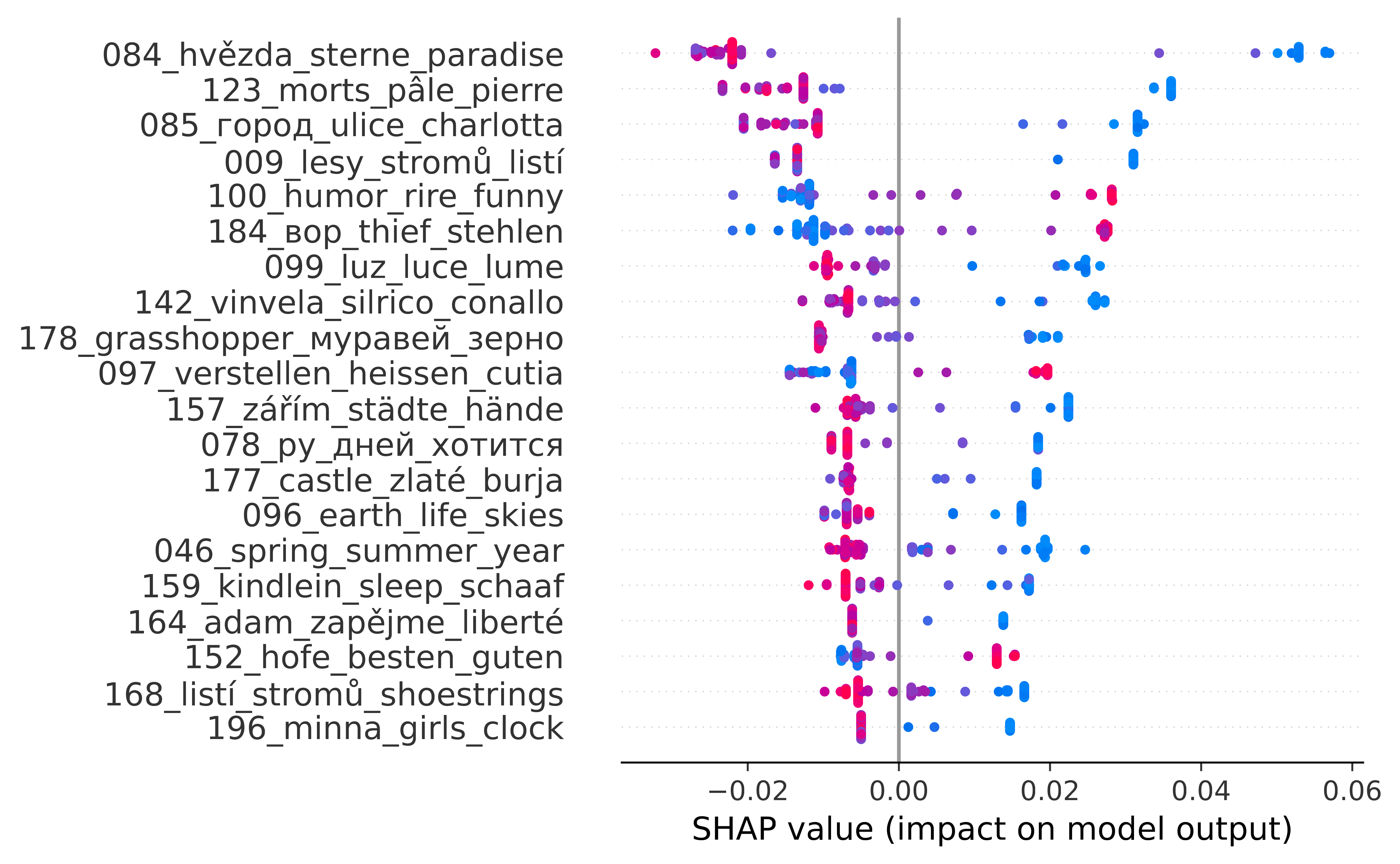

Supplement: S1 Fig — Shapley values in topic-defined vector space. (TIFF) [file pone.0340514.s001.tiff]

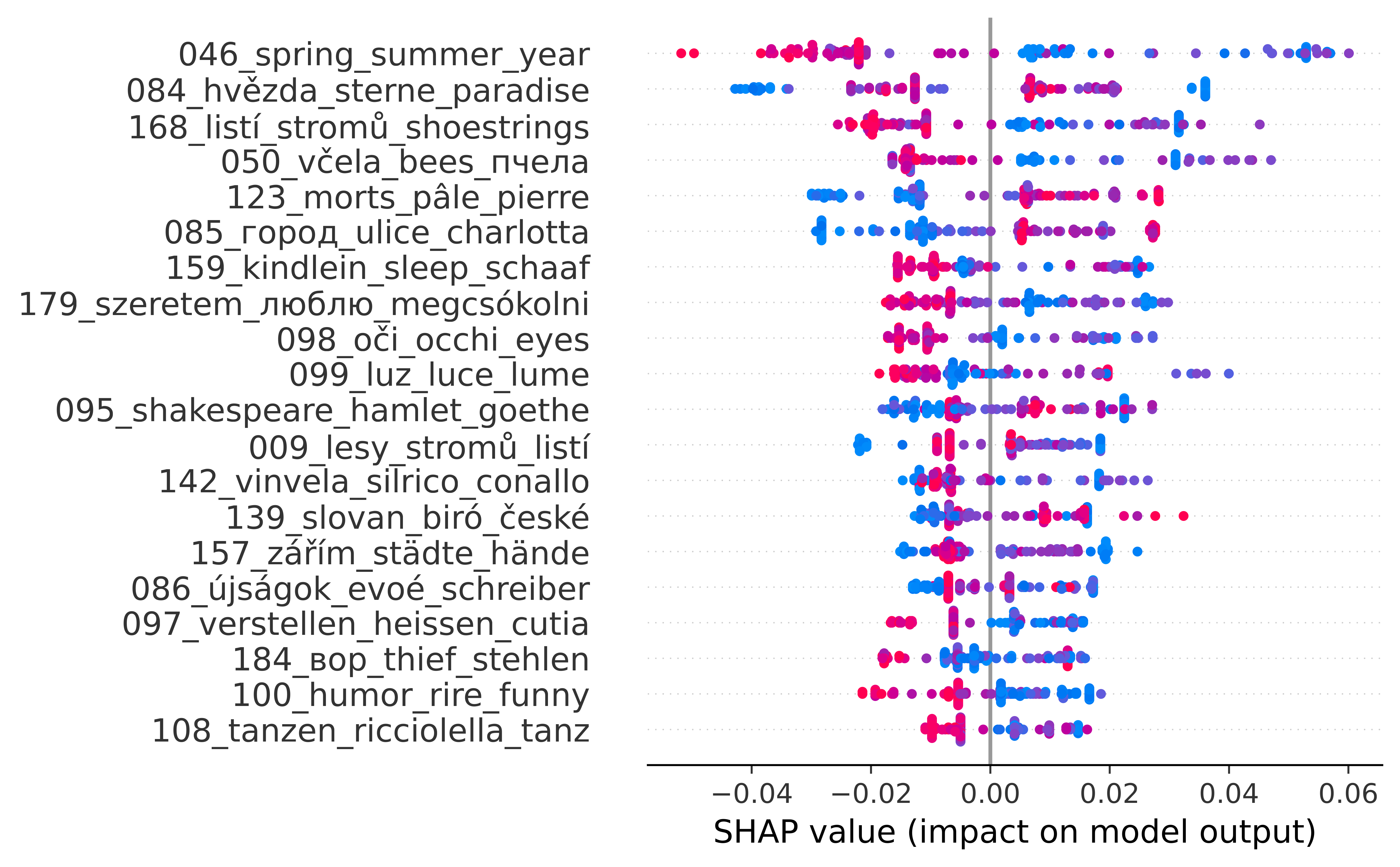

Supplement: S2 Fig — Shapley values in topic-defined vector space. (TIFF) [file pone.0340514.s002.tiff]

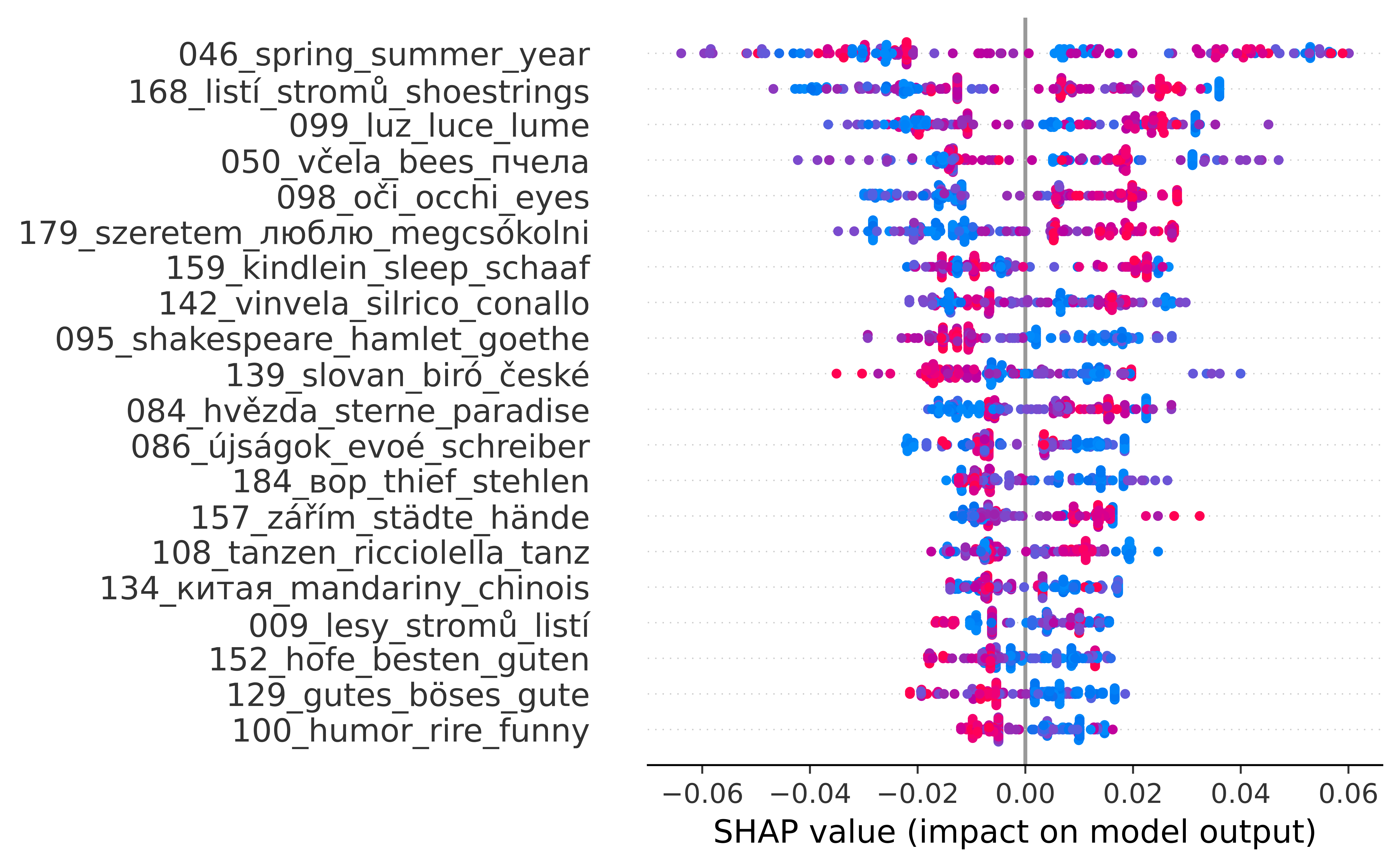

Supplement: S3 Fig — Shapley values in topic-defined vector space. (TIFF) [file pone.0340514.s003.tiff]
