# Supplementary material for: A local sequence alignment approach to recognizing fixed poetic forms across languages
Source: PLoS One. 2026 Jul 14;21(7):e0340514. doi: 10.1371/journal.pone.0340514 (PMC13367689; doi:10.1371/journal.pone.0340514)
Supplement: Attachment — Submitted filename: response.pdf [file pone.0340514.s005.pdf]

We are grateful to the reviewers for the careful and constructive reading of our manuscript *A local sequence alignment approach to recognizing fixed poetic forms across languages*. Below we describe the changes made in response to each point raised by reviewer #2.

1. The reviewer rightly noted that stating the scarcity of large-scale cross-lingual studies, while accurate, does not on its own justify the specific research we undertake. We have revised the introduction to make the stakes of each showcase explicit from the outset.

2. We have added explicit justifications for each of the key methodological decisions.

3. The reviewer points to a genuine gap: our definition of fixed forms as "rhyme patterns reused over time and traditions" does not engage with the substantial theoretical literature on how forms become conventionalized symbols, and the conditions under which that happens. We have expanded the introduction to address this directly.

4. We have added a side-by-side illustration in the Rhyme Encoding section, showing Shakespeare's Sonnet 18 encoded first in conventional rhyme notation and then in our ad hoc scheme, line by line.

5. The duplicate opening paragraph in the Forms Detection section has been removed.

6. We have restructured the relevant paragraphs in the Evaluation section to present the argument in a cleaner sequence.

7. The reviewer asks what happens once a form's symbolic association is secure: does that convention invite experimentation and play? This is an excellent question, and one our data can gesture toward even if a full treatment lies beyond the scope of the current paper. We have added a passage to the discussion that takes up this implication.
