# Supplementary material for: A local sequence alignment approach to recognizing fixed poetic forms across languages
Source: PLoS One. 2026 Jul 14;21(7):e0340514. doi: 10.1371/journal.pone.0340514 (PMC13367689; doi:10.1371/journal.pone.0340514)
Supplement: Peer review history

# Peer review: Decision Letter 0

**DOI:** 10.1371/journal.pone.0340514.r001

**Authors:** Filipi Silva

1 Apr 2026

PONE-D-25-67865

A local sequence alignment approach to recognizing fixed poetic forms across languages

PLOS One

Dear Dr. Plecháč,

Thank you for submitting your manuscript to PLOS ONE. After careful consideration, we feel that it has merit but does not fully meet PLOS ONE’s publication criteria as it currently stands. Therefore, we invite you to submit a revised version of the manuscript that addresses the points raised during the review process.

Please submit your revised manuscript by May 16 2026 11:59PM. If you will need more time than this to complete your revisions, please reply to this message or contact the journal office at plosone@plos.org. When you're ready to submit your revision, log on to https://www.editorialmanager.com/pone/ and select the 'Submissions Needing Revision' folder to locate your manuscript file.

Please include the following items when submitting your revised manuscript:

- A letter that responds to each point raised by the academic editor and reviewer(s). You should upload this letter as a separate file labeled 'Response to Reviewers'.
- A marked-up copy of your manuscript that highlights changes made to the original version. You should upload this as a separate file labeled 'Revised Manuscript with Track Changes'.
- An unmarked version of your revised paper without tracked changes. You should upload this as a separate file labeled 'Manuscript'.

If you would like to make changes to your financial disclosure, please include your updated statement in your cover letter. Guidelines for resubmitting your figure files are available below the reviewer comments at the end of this letter.

If applicable, we recommend that you deposit your laboratory protocols in protocols.io to enhance the reproducibility of your results. Protocols.io assigns your protocol its own identifier (DOI) so that it can be cited independently in the future. For instructions see: https://journals.plos.org/plosone/s/submission-guidelines#loc-laboratory-protocols. Additionally, PLOS ONE offers an option for publishing peer-reviewed Lab Protocol articles, which describe protocols hosted on protocols.io. Read more information on sharing protocols at https://plos.org/protocols?utm_medium=editorial-email&utm_source=authorletters&utm_campaign=protocols.

As the corresponding author, your ORCID iD is verified in the submission system and will appear in the published article. PLOS supports the use of ORCID, and we encourage all coauthors to register for an ORCID iD and use it as well. Please encourage your coauthors to verify their ORCID iD within the submission system before final acceptance, as unverified ORCID iDs will not appear in the published article. *Only*  the individual author can complete the verification step; PLOS staff *cannot*  verify ORCID iDs on behalf of authors.

We look forward to receiving your revised manuscript.

Kind regards,

Filipi N. Silva

Academic Editor

PLOS One

Journal Requirements:

When submitting your revision, we need you to address these additional requirements.

1. Please ensure that your manuscript meets PLOS ONE's style requirements, including those for file naming. The PLOS ONE style templates can be found at

https://journals.plos.org/plosone/s/file?id=wjVg/PLOSOne_formatting_sample_main_body.pdf and

https://journals.plos.org/plosone/s/file?id=ba62/PLOSOne_formatting_sample_title_authors_affiliations.pdf.

2. Please note that PLOS One has specific guidelines on code sharing for submissions in which author-generated code underpins the findings in the manuscript. In these cases, we expect all author-generated code to be made available without restrictions upon publication of the work. Please review our guidelines at https://journals.plos.org/plosone/s/materials-and-software-sharing#loc-sharing-code and ensure that your code is shared in a way that follows best practice and facilitates reproducibility and reuse.

3. Thank you for stating in your Funding Statement:

“PP and AŠ were supported by the Czech Science Foundation, project ga23-07727S (https://gacr.cz/). Funder did not play any role in the study design, data collection and analysis, decision to publish, or preparation of the manuscript.”

Please provide an amended statement that declares *all* the funding or sources of support (whether external or internal to your organization) received during this study, as detailed online in our guide for authors at http://journals.plos.org/plosone/s/submit-now.  Please also include the statement “There was no additional external funding received for this study.” in your updated Funding Statement.

Please include your amended Funding Statement within your cover letter. We will change the online submission form on your behalf.

4. If the reviewer comments include a recommendation to cite specific previously published works, please review and evaluate these publications to determine whether they are relevant and should be cited. There is no requirement to cite these works unless the editor has indicated otherwise.

5. Please review your reference list to ensure that it is complete and correct. If you have cited papers that have been retracted, please include the rationale for doing so in the manuscript text, or remove these references and replace them with relevant current references. Any changes to the reference list should be mentioned in the rebuttal letter that accompanies your revised manuscript. If you need to cite a retracted article, indicate the article’s retracted status in the References list and also include a citation and full reference for the retraction notice.

Additional Editor Comments (if provided):

The reviewers provided positive feedback about your paper. Thus, I'm recommending a minor revision based on Reviewer 2 comments.

[Note: HTML markup is below. Please do not edit.]

Reviewers' comments:

Reviewer's Responses to Questions

**Comments to the Author**

1. Is the manuscript technically sound, and do the data support the conclusions?

The manuscript must describe a technically sound piece of scientific research with data that supports the conclusions. Experiments must have been conducted rigorously, with appropriate controls, replication, and sample sizes. The conclusions must be drawn appropriately based on the data presented.

Reviewer #1: Yes

Reviewer #2: Yes

**********

2. Has the statistical analysis been performed appropriately and rigorously?

Reviewer #1: Yes

Reviewer #2: Yes

**********

3. Have the authors made all data underlying the findings in their manuscript fully available?

The PLOS Data policy requires authors to make all data underlying the findings described in their manuscript fully available without restriction, with rare exception (please refer to the Data Availability Statement in the manuscript PDF file). The data should be provided as part of the manuscript or its supporting information, or deposited to a public repository. For example, in addition to summary statistics, the data points behind means, medians and variance measures should be available. If there are restrictions on publicly sharing data—e.g. participant privacy or use of data from a third party—those must be specified.

Reviewer #1: Yes

Reviewer #2: Yes

**********

4. Is the manuscript presented in an intelligible fashion and written in standard English?

PLOS ONE does not copyedit accepted manuscripts, so the language in submitted articles must be clear, correct, and unambiguous. Any typographical or grammatical errors should be corrected at revision, so please note any specific errors here.

Reviewer #1: Yes

Reviewer #2: Yes

**********

5. Review Comments to the Author

Please use the space provided to explain your answers to the questions above. You may also include additional comments for the author, including concerns about dual publication, research ethics, or publication ethics. (Please upload your review as an attachment if it exceeds 20,000 characters)

Reviewer #1: This is a very publishable paper for a number of excellent reasons. 1. It uses a well-established and already well-publicized open access collection of texts 2. It deals with multilingual texts, cleverly transcending the usually rigid linguistic divide in stylometry. 3. The research is done on poetry and in languages that do not often appear in mainstream and English-dominated research. 4. The statistical methods employed are sound and well-applied. All in all, it is an excellent example of quantitative work on literary texts done by people who know their coding, their statistics and their poetry.

Reviewer #2: This paper introduces a language-independent method for identifying fixed poetic forms across a large multilingual corpus. The approach is unsupervised: rhyme schemes are encoded in a compact symbolic alphabet, pairwise distances are computed using local sequence alignment, and the resulting space is clustered with HDBSCAN after dimensionality reduction with UMAP. The method's utility is illustrated through three downstream analyses. The paper is generally well-written and the results are solid. I have a number of concerns, however, that I think need to be addressed before publication.

1. The paper opens by stating that fixed forms are an important feature of European literary traditions and that large-scale cross-lingual studies have been limited. That is true, but it is not a particularly strong reason to do this specific research. Why does it matter that we can identify these forms automatically and at scale? What questions become answerable that were not before? The three showcases give a partial answer, but the motivation would be stronger if it were connected to those questions from the start. I'd encourage the authors to think more carefully about what is actually at stake here, and to say it clearly in the introduction.

2. The paper does not explain why the specific combination of tools was chosen. Why local sequence alignment rather than, say, edit distance or a 'bag-of-symbols' approach? Why HDBSCAN specifically? The same question applies to UMAP: what parameters were used, and why? This is also an issue for the supervised classification experiments. Why KNN, SVM, and Random Forest? These are reasonable choices, but the reader deserves at least a brief justification. Without it, the methodological choices feel arbitrary, and it becomes harder to interpret where the performance comes from.

3. The authors define fixed forms broadly as "rhyme patterns that have been reused over time and traditions," but this definition is doing a lot of work that is never examined. When does a recurring pattern become a fixed form, and what does that transition involve? There is presumably some threshold of frequency and cultural uptake involved. This connects to the interesting but underdeveloped idea that forms carry meaning across traditions. The question of how forms become conventionalized symbols, and under what social and historical conditions that happens, seems central to what the paper is trying to do. Embedding the discussion more firmly in that literature would both strengthen the motivation and make the showcases more meaningful.

4. The ad hoc encoding scheme introduced in the paper is not easy to follow from the text alone. An illustration showing a concrete example side by side with its conventional notation and its new encoding would help a lot. The prose description is a bit dry and I had to read it twice to understand what was going on.

5. In the "Forms detection" section, the first paragraph appears twice. Please remove the duplicate.

6. Maybe this is just me, but the discussion of precision values relative to the LLM-based approach is confusing. The authors note that the evaluation conditions are different, and that restricting evaluation to gold-positive poems makes the comparison much more favorable. After this correction is applied, the differences apparently become small. The way it is currently written, the reader has to do too much work to figure out what the authors are actually claiming.

7. The authors show that Italian-origin forms continue to carry references to Italy in other traditions, and that certain forms have consistent thematic associations across languages. That is a real finding and deserves more attention. But I was left wondering what happens after such an association becomes established. Once readers know that terza rima signals Dante and the Italian Renaissance, does that convention start to invite experimentation? Do later poets play against the expected association precisely because the convention is secure enough to be recognizable? The paper touches the surface of this question but does not pursue it. Even a brief discussion of what conventionalization implies for literary history would make the showcases feel less like proofs of concept and more like genuinely new contributions.

**********

6. PLOS authors have the option to publish the peer review history of their article (what does this mean?). If published, this will include your full peer review and any attached files.

If you choose “no”, your identity will remain anonymous but your review may still be made public.

**Do you want your identity to be public for this peer review?**  For information about this choice, including consent withdrawal, please see our Privacy Policy.

Reviewer #1: **Yes:** Jan Rybicki

Reviewer #2: No

**********

[NOTE: If reviewer comments were submitted as an attachment file, they will be attached to this email and accessible via the submission site. Please log into your account, locate the manuscript record, and check for the action link "View Attachments". If this link does not appear, there are no attachment files.]

To ensure your figures meet our technical requirements, please review our figure guidelines: https://journals.plos.org/plosone/s/figures

You may also use PLOS’s free figure tool, NAAS, to help you prepare publication quality figures: https://journals.plos.org/plosone/s/figures#loc-tools-for-figure-preparation.

NAAS will assess whether your figures meet our technical requirements by comparing each figure against our figure specifications.

# Author comment: Author response to Decision Letter 1

**DOI:** 10.1371/journal.pone.0340514.r002

13 May 2026

1. The reviewer rightly noted that stating the scarcity of large-scale cross-lingual studies, while accurate, does not on its own justify the specific research we undertake. We have revised the introduction to make the stakes of each showcase explicit from the outset.

2. We have added explicit justifications for each of the key methodological decisions.

3. The reviewer points to a genuine gap: our definition of fixed forms as “rhyme patterns reused over time and traditions” does not engage with the substantial theoretical literature on how forms become conventionalized symbols, and the conditions under which that happens. We have expanded the introduction to address this directly.

4. We have added a side-by-side illustration in the Rhyme Encoding section, showing Shakespeare’s Sonnet 18 encoded first in conventional rhyme notation and then in our ad hoc scheme, line by line.

5. The duplicate opening paragraph in the Forms Detection section has been removed.

6. We have restructured the relevant paragraphs in the Evaluation section to present the argument in a cleaner sequence.

7. The reviewer asks what happens once a form’s symbolic association is secure: does that convention invite experimentation and play? This is an excellent question, and one our data can gesture toward even if a full treatment lies beyond the scope of the current paper. We have added a passage to the discussion that takes up this implication.

Attachment Submitted filename: response.pdf

# Editor report: Decision Letter 1

**DOI:** 10.1371/journal.pone.0340514.r003

**Authors:** Filipi Silva, Filipi Silva

15 May 2026

A local sequence alignment approach to recognizing fixed poetic forms across languages

PONE-D-25-67865R1

Dear Dr. Plecháč,

We’re pleased to inform you that your manuscript has been judged scientifically suitable for publication and will be formally accepted for publication once it meets all outstanding technical requirements.

Within one week, you’ll receive an e-mail detailing the required amendments. When these have been addressed, you’ll receive a formal acceptance letter and your manuscript will be scheduled for publication.

An invoice will be generated when your article is formally accepted. Please note, if your institution has a publishing partnership with PLOS and your article meets the relevant criteria, all or part of your publication costs will be covered. Please make sure your user information is up-to-date by logging into Editorial Manager at Editorial Manager® and clicking the ‘Update My Information' link at the top of the page. For questions related to billing, please contact billing support.

If your institution or institutions have a press office, please notify them about your upcoming paper to help maximize its impact. If they’ll be preparing press materials, please inform our press team as soon as possible -- no later than 48 hours after receiving the formal acceptance. Your manuscript will remain under strict press embargo until 2 pm Eastern Time on the date of publication. For more information, please contact onepress@plos.org.

Kind regards,

Filipi N. Silva

Academic Editor

PLOS One

Additional Editor Comments (optional):

Thank you for the careful revisions and clarifications throughout the manuscript. I am happy to recommend the paper for publication.

Reviewers' comments:

# Editor report: Acceptance letter

**DOI:** 10.1371/journal.pone.0340514.r004

**Authors:** Filipi Silva, Filipi Silva

PONE-D-25-67865R1

PLOS One

Dear Dr. Plecháč,

I'm pleased to inform you that your manuscript has been deemed suitable for publication in PLOS One. Congratulations! Your manuscript is now being handed over to our production team.

At this stage, our production department will prepare your paper for publication. This includes ensuring the following:

* All references, tables, and figures are properly cited

* All relevant supporting information is included in the manuscript submission,

* There are no issues that prevent the paper from being properly typeset

You will receive further instructions from the production team, including instructions on how to review your proof when it is ready. Please keep in mind that we are working through a large volume of accepted articles, so please give us a few days to review your paper and let you know the next and final steps.

Lastly, if your institution or institutions have a press office, please let them know about your upcoming paper now to help maximize its impact. If they'll be preparing press materials, please inform our press team within the next 48 hours. Your manuscript will remain under strict press embargo until 2 pm Eastern Time on the date of publication. For more information, please contact onepress@plos.org.

You will receive an invoice from PLOS for your publication fee after your manuscript has reached the completed accept phase. If you receive an email requesting payment before acceptance or for any other service, this may be a phishing scheme. Learn how to identify phishing emails and protect your accounts at https://explore.plos.org/phishing.

If we can help with anything else, please email us at customercare@plos.org.

Thank you for submitting your work to PLOS ONE and supporting open access.

Kind regards,

PLOS ONE Editorial Office Staff

on behalf of

Filipi N. Silva

Academic Editor

PLOS One
